# Unexpected Crossover in the kinetics of mutarotation in the supercooled region: the role of H-bonds

**DOI:** 10.1038/s41598-018-23117-8

**Published:** 2018-03-28

**Authors:** K. Wolnica, M. Dulski, E. Kaminska, M. Tarnacka, R. Wrzalik, W. E Śmiszek-Lindert, K. Kaminski, M. Paluch

**Affiliations:** 1 Silesian Center for Education and Interdisciplinary Research, 75 Pulku Piechoty 1a, 41-500 Chorzow, Poland; 2 A. Chelkowski Institute of Physics, University of Silesia, 75 Pulku Piechoty 1, 41-500 Chorzow, Poland; 3 Institute of Material Science, University of Silesia, 75 Pulku Piechoty 1a, 41-500 Chorzow, Poland; 4 Department of Pharmacognosy and Phytochemistry, Medical University of Silesia in Katowice, School of Pharmacy with the Division of Laboratory Medicine in Sosnowiec, Jagiellonska 4, 41-200 Sosnowiec, Poland

## Abstract

Intra- and intermolecular studies on the molten L-sorbose have been carried out at variable temperature conditions to determine the crosover temperature (*T*_*c*_). In addition, isothermal time-dependent FTIR and Raman measurements were performed to probe the pace of mutarotation and activation energy of this reaction in the studied saccharide, which varied from 53–62 kJ/mol up to 177–192 kJ/mol below and above *T*_*c*_, respectively. To explain the change in activation barrier for the mutarotation a complementary analysis using difference FTIR spectra collected around *T*_*c*_ = 365 K in the hydroxyl region has been done. It was found that the alteration of kinetic parameters and molecular dynamics around *T*_*c*_ are strictly related to the variation in the strength of H-bonds which above *T*_*c*_ are significantly weaken, increasing the freedom of rotation of functional groups and movement of individual molecules. That phenomenon most likely affects the proton transfer, underlying molecular mechanism of mutarotation, which may lead to the significant increase in activation barrier. The new insight into a molecular aspect of the mutarotation around *T*_*c*_ has created an opportunity to better understanding the relationship between physics of condensed matter and the potential role of H-bonds dynamics on the progress of the chemical reaction in highly viscous systems.

## Introduction

The phenomenon of crossover temperature (*T*_c_) in molecular dynamics, that in common view marks the onset of the cooperative dynamics, has been studied intensively in the past. The first reports touching this issue began in the ‘60 s when Goldstein postulated that the change in dynamics from the liquid like to viscous flow driven by over-barrier relaxation should occur around *τ*_*α*_ ≈ 10^−9^ s^1^. Further studies by Novikov *et al*.^2^ have shown that for most systems, including those that are covalent, hydrogen bonding, ionic, molecular, polymeric, the crossover temperature (*T*_*c*_ ≈ 1.2 *T*_*g*_) corresponds roughly to the structural relaxation times *τ*_*α*_ ≈ 10^−7^–10^−8^. Similar conclusions were also derived by Boyer in case of polymers. The authors claimed that at higher temperature there might be the 3^rd^ order liquid to liquid transition^3,4^. It should be stressed that very important progress in this field was done with the development of the Mode-Coupling Theory (MCT)^5,6^ that provides a quantitative description of the dynamics of simple dense liquids around the triple point as well as the cage dynamics. One can mention that this theory predicts the existence of critical temperature (*T*_*c*_), where the change from a liquid-like to solid-like dynamics occurs. Interestingly, further numerous studies with the use of various experimental techniques carried out on different materials showed unquestionably that *T*_*c*_ is rather a universal feature of supercooled liquids^7–10^. It was also found that in the vicinity of this point, there is a decoupling between various physical quantities, such as conductivity, diffusion, viscosity or structural relaxation times^11–15^. Moreover, in many cases, the splitting of the global and local relaxation processes identified as structural and secondary relaxation modes is noted. In this context, one can recall papers by Kremer *et al*. who investigated the dynamics of simple glass formers by means of FTIR and BDS spectroscopies^16–18^. They demonstrated that in the temperature range (*T* ≈ 1.2 *T*_*g*_), where the structural and local processes merge, a clear change in intramolecular mobility can be detected.

**Figure 1:**
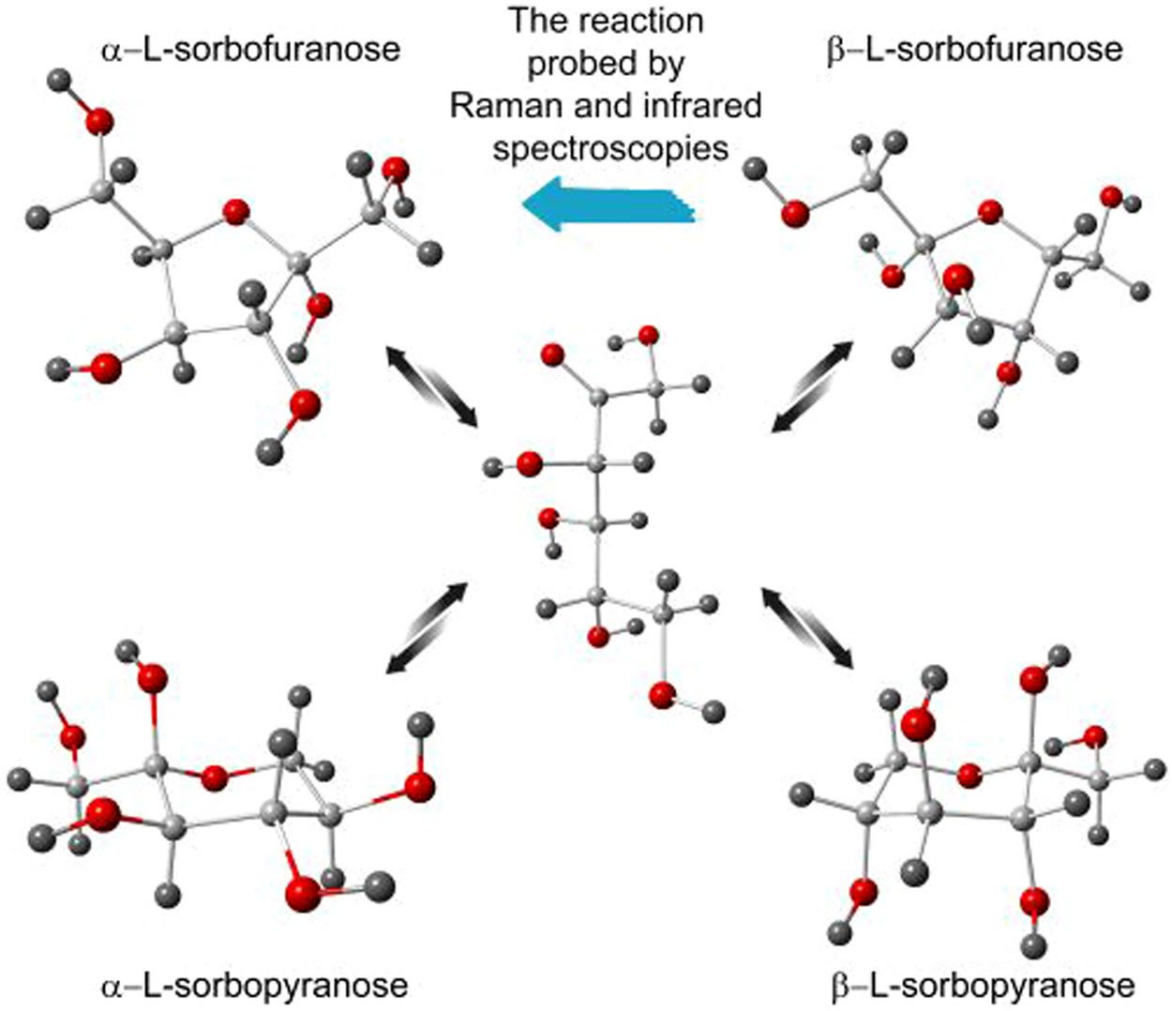
The various possible isomers of L-sorbose appearing during the mutarotation. The linear conformation is a transitional state with negligible lifetime and concentration at any time scale of the experiments. The color of balls is corresponding to suitable atoms: red: oxygen, light gray: carbon, dark gray: hydrogen.

Interestingly, over the years only physical and dynamical properties of the soft matter around *T*_*c*_ have been mainly studied. On the other hand, the impact of crossover in dynamics (*T*_*c*_) on the progress of chemical reactions has not been tested so far. It was related to the fact that generally reactions are carried out in a solvent, in systems of low viscosity, to make any chemical conversion very fast and effective. The main motivation of this work is to fill that gap and study the progress of chemical conversion around the crossover temperature. For that purpose, a model chemical reaction, mutarotation in L-sorbose, has been investigated around *T*_*c*_. Briefly, mutarotation is due to the equilibration between various forms of the analyzed saccharide, such as α, β - pyranose (hexose), α, β -furanose (pentose) or open chain conformations (Fig. 1). The reaction begins as soon as the crystal structure of given saccharide is destroyed.

Although the progress and kinetics of this reaction have been studied since 1846, when Dubrunfaut^19^ discovered it, still, new data and experimental finding are coming out that contribute to revisiting current models describing mutarotation. In this context one can remind very recent studies on pure supercooled saccharides demonstrating that the progress of mutarotation in solid state is much different than the kinetics of the reaction carried out in various solvents^20–26^. It is well-manifested in the two times higher activation barrier and sigmoidal shape of the kinetic curves^21^. Such differences are linked to the various mechanisms of the reaction which are probably strongly correlated with the structural modification related to the inter- and intramolecular H-bond scheme, further affecting the proton transfer in the system.

To investigate the progress of mutarotation in the solid state around *T*_c_, FTIR, Raman, and broadband dielectric spectroscopies were used^27–29^. It is worth to mention that polarimetry, which is well established historical method to study mutarotation fails and can not be applied to probe the progress of this chemical conversion in the supercooled highly viscous regime. Application of mentioned above methods enabled us to determine the crossover temperature, dynamics of L-sorbose, progress of mutarotation, as well as analysis of the H-bond pattern above and below *T*_c_ in this saccharide. Combination of the data obtained from various techniques allowed to assign unexpected change in the kinetics of mutarotation around *T*_c_ to the variation in dynamics and strength of H-bonds.

## Results and Discussion

**Figure 2:**
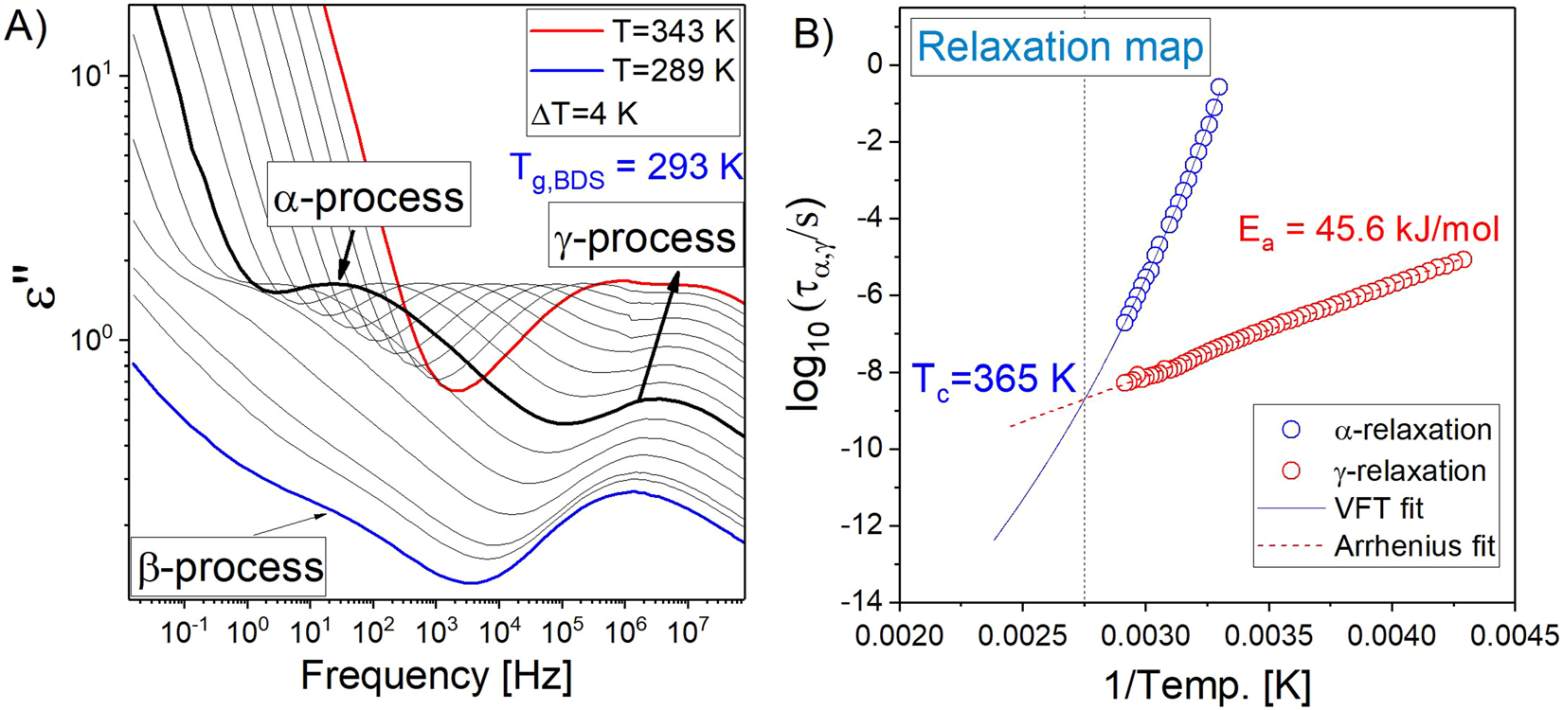
(**A**) Dielectric loss spectra measured for L-sorbose in the vicinity and above *T*_*g*_. (**B**) Temperature dependence of the structural (*α*) and secondary (*γ*) relaxation times. Solid and dashed lines represent VFT and Arrhenius fits, respectively.

Dielectric loss spectra obtained for neat molten L-sorbose at various temperatures are presented in Fig. 2. Above *T*_*g*_, one can observe the structural or α-relaxation process assigned to the cooperative motions of molecules, which is considered to be responsible for the viscous flow and the liquid-to-glass transition. This process is generally characterized by the non-Arrhenius temperature dependence of relaxation times above *T*_*g*_.

In the glassy state (below *T*_*g*_), additional relaxation processes can be detected, termed as secondary relaxations (usually named as *β*, *γ*, *δ*, …), referring to different local motions of dipole active parts of molecules. In L-sorbose one can see two secondary processes (*β* and *γ*). The slower one (*β*) (not analyzed herein) is visible as an excess wing on the high-frequency tail of the α-relaxation, while at higher frequencies a well resolved *γ*-loss peak appears. Recently, molecular origin of this process has been identified to be associated with librational motions of exocyclic methylene units^30^.

In order to evaluate the dynamics of *α-* and *γ*-relaxation processes in L-sorbose, the dielectric loss spectra were fitted to the superposition of two Havriliak-Negami (HN) functions^31^ with an additional term describing the dc conductivity contribution (see Fig. 2A):

1 $$\varepsilon (\varpi )^{\prime\prime} =\frac{{\sigma }_{dc}}{{\varepsilon }_{0}\varpi }+\frac{{\rm{\Delta }}\varepsilon }{{(1+{(i\varpi {\tau }_{HN})}^{\alpha })}^{\beta }}+{\varepsilon }_{{\rm{\infty }}}$$

where *α* and *β* are the shape parameters representing the symmetric and asymmetric broadening of given relaxation peaks, Δ*ε* is the dielectric relaxation strength, *τ*_*HN*_ is the HN relaxation time, and ϖ is an angular frequency (*ϖ* = *2πf*). Then, using well-known protocol^32^, *τ*_*α*_ and τ_γ_ were calculated from *τ*_*HN*_ and plotted vs reciprocal temperature in Fig. 2B. Next, we used the Vogel-Fulcher-Tammann (VFT) and Arrhenius equations to fit the dependences of the structural relaxation time of alpha, *τ*_*α*_(*T*), and gamma, *τ*_*γ*_(*T*), processes, respectively. The applied VFT function is defined as follows:

2 $${\tau }_{\alpha }={\tau }_{{\rm{\infty }}}\exp (\frac{{D}_{T}{T}_{0}}{T-{T}_{0}})$$

where *τ*_*α*_*, D*_*T*_ and *T*_0_ are parameters determined by fitting to the experimental data. Consequently, both the glass transition temperature (*T*_*g*_ = 293 K) and activation barrier (*E*_*A*_ = 45.6 kJ/mol) for the *γ*-relaxation process were calculated. It should be noted that *T*_*g*_ was defined as a temperature at which structural relaxation time is equal to 100 s. Interestingly, similar values of the glass transition temperatures and the activation barriers for the secondary relaxation have been reported for other monosaccharides^33^.

We wish to emphasize again that *α* and *γ* relaxation processes tend to merge at some temperature, as it can be observed in Fig. 2. By the extrapolation of the Arrhenius fits to higher temperatures, one can estimate the value of splitting temperature (*T*_*c*_ = 365 K). Interestingly, this temperature corresponds to the structural relaxation time log *(τ*_*α*_ *)* ≈ −9.00. Accordingly to the data reported for various glass formers, indicating *T*_*c*_ to occur within the range between *τ*_*α*_ = 10^−7^–10^−9^ (log*(τ*_*α*_*)* ≈ −7.00 to −9.00), one can suppose that we found a crossover in dynamics of L-sorbose.

**Figure 3:**
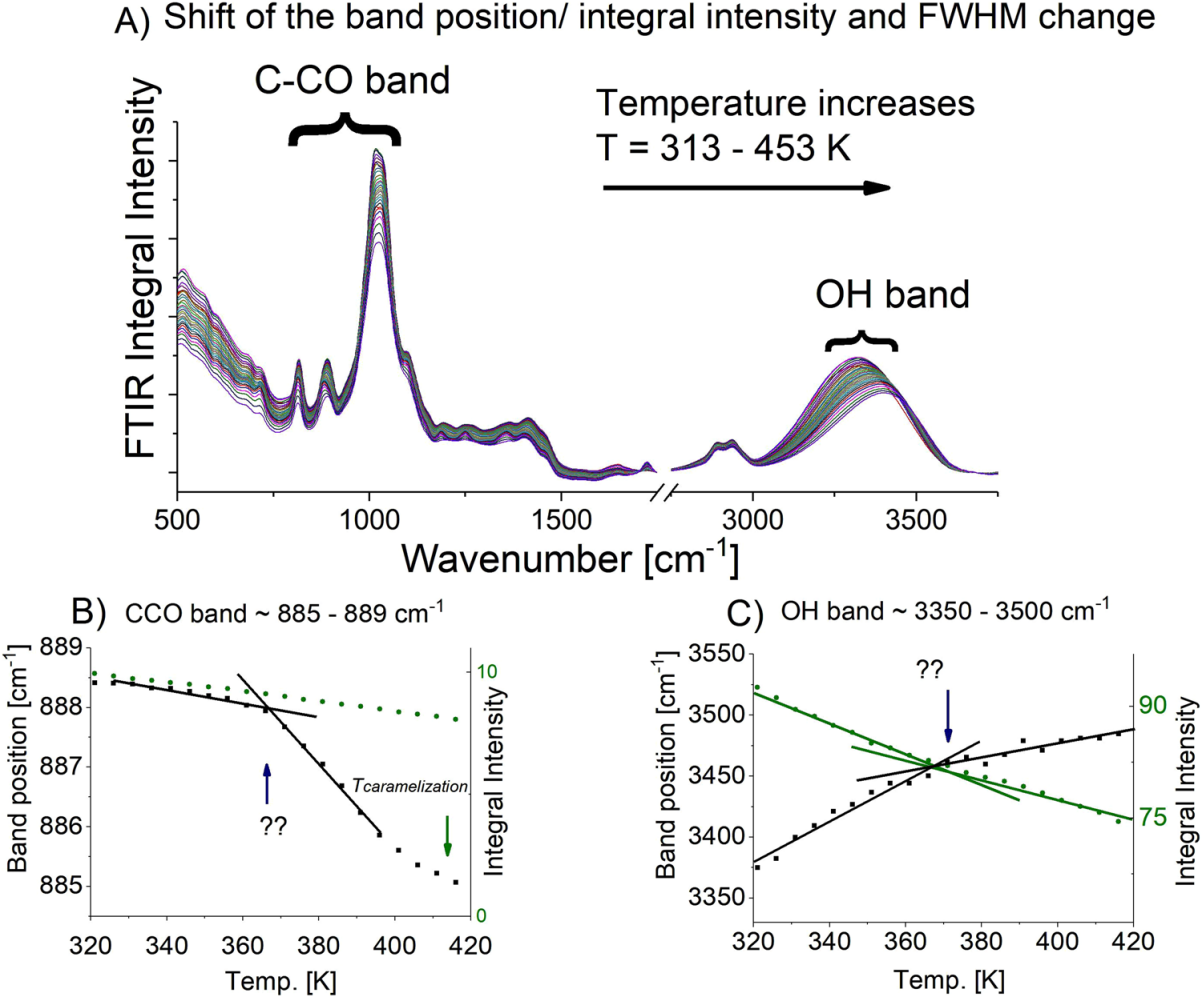
(**A**) FTIR spectra of L-sorbose measured in the temperature range *T* = 313 ÷ 453 K. Clear changes in a peak position ascribed to the OH group and C-CO bands can be observed. (**B**,**C**) Changes of CCO and OH band positions as well as integral intensities versus temperature. Points of characteristic changes have been marked with the blue arrow at around *T* = 365 K and with the green arrow at around *T* = 415 K (the temperature of saccharide’s caramelization).

Recent reports by the group of Kremer^16–18^ also demonstrated that the splitting of the structural and secondary relaxation processes at *T*_*c*_ is very often accompanied by the change in intramolecular dynamics. Therefore, to address this issue additional temperature-dependent FTIR measurements above and below *T*_*c*_ were carried out to follow intramolecular dynamics in L-sorbose. As can be seen in Fig. 3A–C, the position, and intensity of some bands in the studied herein carbohydrate are permanently changing with the increase of temperature. To explain this more precisely, theoretical calculations were performed to assign the vibrations of individual fragments to the bands observed in the experimental infrared spectrum. Considering band assignments and data reported previously by Kossack *et al*.^28^ or Dulski *et al*.^34^ for other monosaccharides, i.e., L-fucose or D-fructose, a few experimental bands obtained due to the fitting of the infrared spectrum using the Voigt function, have been found. They are assigned to the vibrational modes of OH (ν_(OH)_ = 3375 cm^−1^) and CCO groups within the carbohydrate rings (ν_(CCO)_ = 1036, 885, 815 cm^−1^), which in view of the paper by Kossack *et al*.^28^ provide the most important information about intramolecular dynamics in saccharides^26^. It is worth to note that due to overlapping of the band at 1036 cm^−1^ with the other ones we carried out analysis of the bands at 885 and 815 cm^−1^ which are well-separated and may be assigned mainly to only one type of vibration. The integral intensities (*I*), as well as their position (*υ*_*max*_) are changing significantly with the temperature. More precisely, the bands at 885 and 815 cm^−1^ are shifted towards lower wavenumbers, while the band at 3375 cm^−1^ is shifted towards higher wavenumbers with increasing temperature (Fig. 3). Integrated intensity values for CCO bands returned similar characteristics, wherein the opposite trend was found in the case of the OH band. To explain this behavior, Kossack *et al*.^28^ have reported that CCO bonds may tend to shorten probably due to stronger molecular interaction or atom rearrangement within the saccharide ring. In turn, disrupting of the inter- and intramolecular H-bonding pattern results from the increase of the distance between oxygen and hydrogen atoms upon temperature growth (Fig. 3). One can add that integrated intensity (*I*) values of each analyzed band decrease with increasing temperature, originating from the system dynamics and molecular rearrangement. What is more, the analysis of *υ*_*max*_ and *I* revealed more or less visible kink in temperature dependences at *T* ≈ 365 K, indicating a change in the dynamics in L-sorbose resulted from inter- and intramolecular rearrangement. This temperature corresponds very well to the *T*_*c*_ evaluated from previously obtained dielectric data. Similar observations, but without special explanation, have been also reported by Kaminski *et al*.^35^ and the group of Kremer^17,18^. Such correspondence between intra- and intermolecular dynamics was found much above the glass transition temperature (*T* ≈ 1.2 *T*_g_). Therefore, this is further evidence that around *T* = 365 K there is a crossover in dynamics of L-sorbose.

Having in mind the results of inter- and intramolecular investigations performed with the use of BDS and FTIR techniques, we decided to examine experimentally the progress of mutarotation in the supercooled state, both above and below the *T*_*c*_. Recently, many articles on the monitoring of mutarotation in the vicinity of the glass transition temperature with the use of dielectric spectroscopy have been published^21–24,26,36^. In these papers, it was reported that the changes in dielectric spectra get much smaller and are barely detectable with increasing temperature. Furthermore, a reaction becomes so fast that BDS method cannot be used to monitor it. Therefore, to overcome this problem we applied FTIR and Raman spectroscopies to follow the kinetics of this canonical chemical conversion. It is worth adding that Kossack *et al*.^28^, Dulski *et al*.^34^ and Dujardin *et al*.^27^ demonstrated separately that these techniques are capable to study mutarotation process in the supercooled state in highly viscous saccharides.

Therefore, a systematic infrared and Raman measurements have been carried out to study the progress of mutarotation in L-sorbose around *T*_*c*_. Although both spectroscopic methods yielded complementary information and returned very similar results on the progress of mutarotation, several clear advantages of Raman technique over Infrared one appeared. The first one is related to the time of accumulation of a single spectrum and some delay appearing during the data acquisition in case of the latter technique. On the other hand, Raman spectroscopy enables much faster data acquisition due to the implementation of the fast scan option in the software. Hence, infrared spectroscopy provides data collection in the second time-scale while Raman technique ensures millisecond time of acquisition of a single spectrum. Additionally, it should be mentioned that the bands in the Raman spectrum are much better separated from each other. Consequently, assignments of molecular vibration to a given band and the analysis of a variety of integral intensities are much easier to perform. Mainly due to the discussed reasons, in the further part of this paper, a greater attention will be focused on the results obtained from Raman measurements.

**Figure 4:**
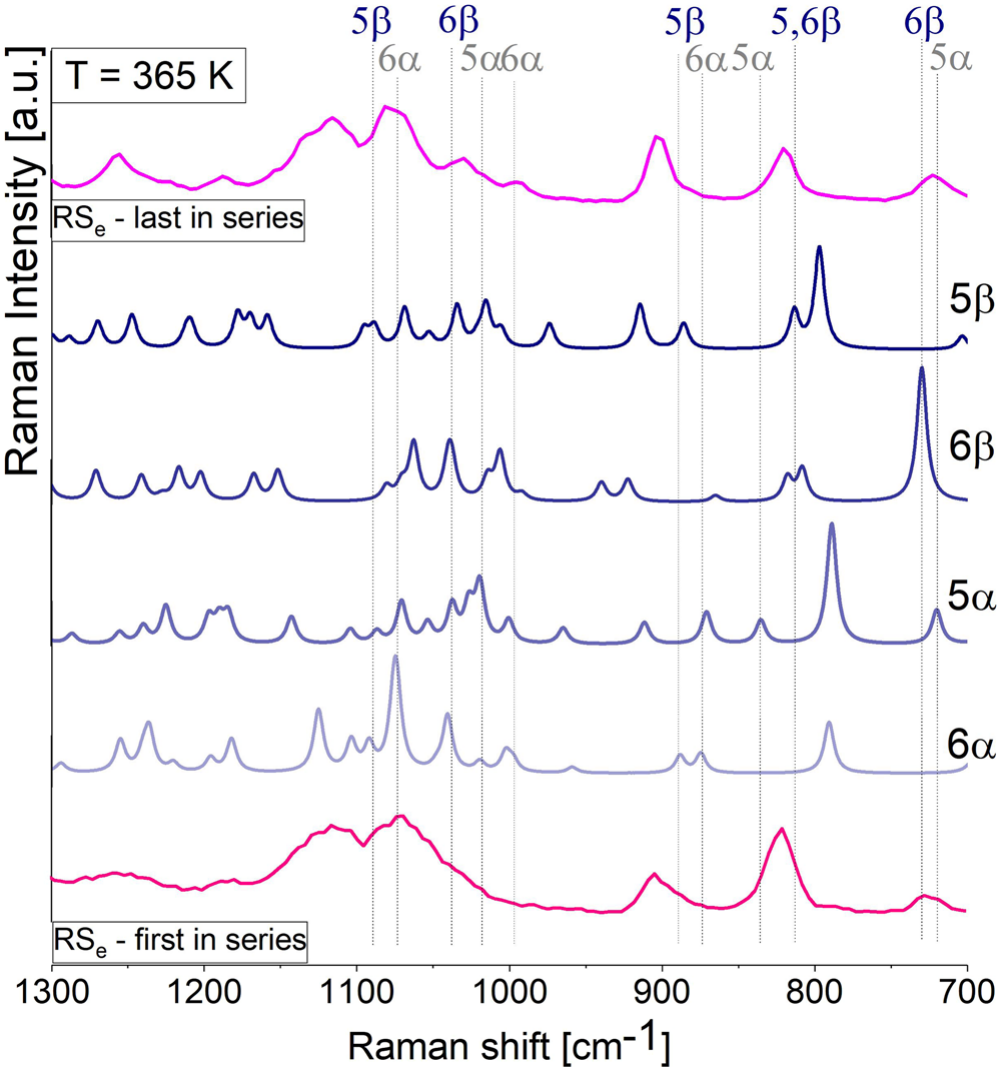
Comparison of experimental Raman spectra of molten L-sorbose in the region of 1300–700 cm^−1^ (dark pink for the first spectrum in series and pink for the last spectrum in series) measured at ambient temperature with theoretical data calculated for α, β - sorbofuranose and α, β - sorbopyranose isomers obtained at DFT/B3LYP/6–311++ (2d, 2p) levels of theory (blue lines).

Hence, similar to FTIR investigations, the experimental Raman spectra were compared to the ones obtained from theoretical calculations – Fig. 4 (the same procedure was performed by Wolnica *et al*. for D-fructose^29^). This simple approach enabled to identify the bands originating from the vibrations occurring within various isomers of L-sorbose. One can find that bands at 726/790, 903/876, 994/981, 1080/1036 cm^−1^ (Raman/FTIR) and 822/815, 910/900, 1010/1006, 1020/1011 cm^−1^ (Raman/FTIR) may originate from the vibration of either α- or β- L-sorbofuranose or L-sorbopyranose, respectively (Table 1). However, according to greater or smaller contribution of various molecular configurations, it is difficult to find only one experimental single marker band that can be assigned to solely one 5- or 6-membered ring isomer of this saccharide^29^. On the other hand, the correlation between theoretical and experimental assignments allowed tracking the conversion between α and β isomers, while the transformation between pyranose and furanose was subjected to a too high degree of uncertainty and required further confirmation by other techniques (Fig. 4). For greater certainty, additional calculations on four different systems composed of various populations of α and β isomers of sorbofuranose and sorbopyranose have been carried out (data not shown). The great advantage of this method was a determination of the concentration of given isomer of L-sorbose and reconstruction of the evolution of experimental spectrum measured during the reaction. At first glance, no significant change in the population of furanose and pyranose forms of L-sorbose during the reaction in the studied temperature range has been observed. However, theoretical integral intensities (*I*) associated to vibrational modes of CC bond of β isomer (bands at 822, 1020 cm^−1^) are slightly higher than those of α isomer (bands at 903, 994 cm^−1^) at the beginning of the process and tend to change with reaction. A similar observation has been found for the infrared analysis and bands centered at 815, 1011 cm^−1^ (β isomer) and at 876, 981 cm^−1^ (α isomer). Comparison between experimental Raman or infrared spectra obtained at different times of mutarotation with the ones calculated theoretically for various models enabled to detect the decrease and increase of integral intensities of bands of β- and α- isomers suggesting β-isomer consumption upon mutarotation.

**Table 1:** Theoretical assignments to the bands observed on the FTIR and Raman spectra presented in Fig. 5, where individual nature of vibrational mode was depicted as ν - stretching, δ - deformation; (CCO)* refers to the saccharide ring^47^.

ν_FTIR_ [cm^−1^]	ν_RS_ [cm^−1^]	Theoretical band assignments			
		α-L-sorbopyranose	β-L-sorbopyranose	α-L-sorbofuranose	β -L-sorbofuranose
790	726	ν(CCO)*, δ(CH_2_)	δ(OH), δ(CH_2_)	ν(CCO)*, δ(CH),	
815	822		ν(CCO)*, δ(CH),	δ(CH), δ(OH)	ν(CCO)*, δ(CH),
876	903	δ(CH_2_), δ(OH)		ν(CCO)*, δ(CH), δ(CH_2_)	
900	910			ν(CC), δ(CH), δ(CH_2_)	ν(CCO)*, δ(CH_2_), δ(OH)
930	949		ν(CCO)*, δ(CH_2_), δ(OH)	δ(CH), δ(CH_2_)	
981	994	ν(CCO)*, δ(CH), δ(OH)	δ(CH_2_), δ(OH)		
1006	1010	δ(CH_2_), δ(OH)		ν(CCO)*, δ(CH_2_), δ(OH)	
1011	1020		ν(CCO)*, δ(OH)		ν(CCO)*, δ(CH_2_), δ(OH)
1036	1080	ν(CCO)*, δ(OH)	δ(CH_2_), δ(OH)	δ(CH_2_), δ(OH)	δ(CH_2_), δ(OH)
3322	3380	ν(OH)	ν(OH)	ν(OH)	ν(OH)

**Figure 5:**
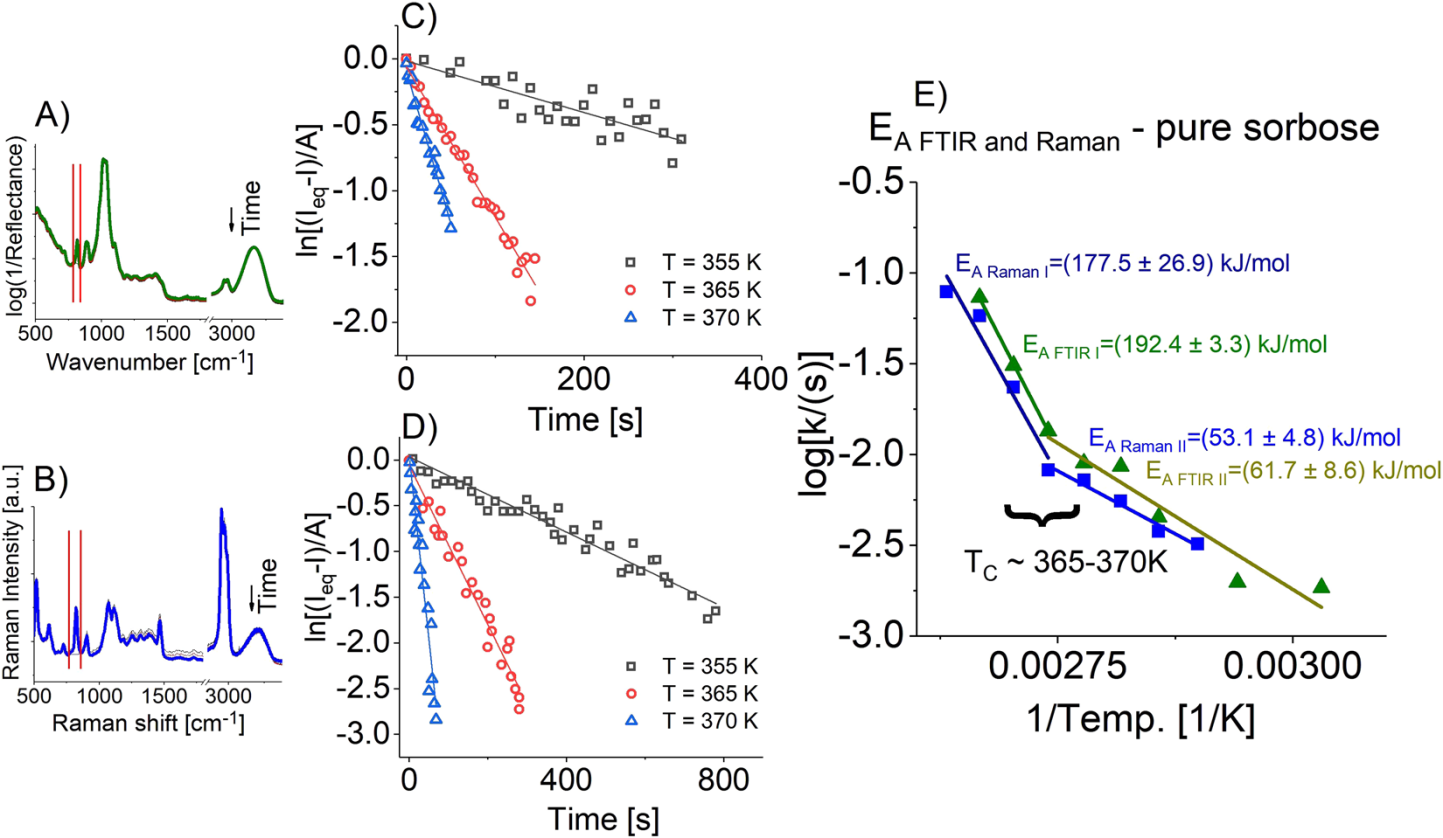
Representation of time-depended evolution of pure L-sorbose infrared (**A**) and Raman (**B**) spectra with markers (red solid lines) of band originating from α-ring vibration (υ_FTIR_ = 815 cm^−1^, υ_Raman_ = 822 cm^−1^) of L-sorbose. Determination of constant rates at different temperatures by plotting the time dependence of ln(*I*_*eq*_ − *I*)*/A*, where *I*_*eq*_ is the final (equilibrium) value of integral intensity from plateau region of kinetics curves, *I* is the integral intensity at a given time and *A* is the amplitude of the *I* signal for single band in the case of infrared (**C**) and Raman (**D**) studies. All data were re-scaled according to the procedure adopted from Wolnica *et al*.^29^. (**E**) Time dependence of log(*k*) obtained from FTIR (green triangles) and Raman (blue squares). Solid lines represent Arrhenius fits. Obtained activation barriers for mutarotation of pure L-sorbose with standard deviations were also included.

Taking into account above considerations, the isothermal kinetics curves obtained from the time-dependent analysis of integrated intensity of well-separated band at 822 cm^−1^ (Raman) or at 815 cm^−1^ (FTIR) (Fig. 5A,B) for the measurements carried out in the following temperature range: 330–380 K have been constructed. Interestingly, they follow exponential character characteristic for the first-order reactions (data not shown). The data were further fitted using the first-order kinetic equation:

3 $$I=A\,\exp \,(-kt)+C$$

where *I* is a given integral intensity, *k* is a constant rate and *C* is a constant. Additionally, so-obtained results were recalculated using the formula given as ln[(*I*_*eq*_ − *I*)*/A*] and plotted *vs*. time, where *I, I*_*eq*_ and *A* denote the integral intensity at given time of reaction, the equilibrium value of *I* from plateau region and the amplitude of *I* variation, respectively. Presented dependencies that can be well described by a linear function with the slope identified as a constant rate (*k*) of consumption of β isomer of L-sorbose (see Fig. 5C,D) confirmed the first-order kinetic character of the process.

Next, log(*k*) were plotted as a function of inverse temperature in Fig. 5E and fitted to the Arrhenius equation to calculate the activation barrier of the mutarotation reaction:

4 $$k={k}_{0}\exp (\frac{{E}_{A}}{RT})$$

where *k*_0_ is a pre-exponential factor, *E*_*A*_ is an activation energy and *R* is the universal gas constant. The rates determined from the analysis of integral intensities of FTIR and Raman bands are depicted in Fig. 5E. As can be seen, there is a clearly visible kink in the temperature dependence of log(*k*). Interestingly, such change in constant rates is observed at temperature *T* ≈ 365 K that corresponds to the *T*_*c*_ which was earlier determined from the inter- and intramolecular investigations with the use of BDS and FTIR spectroscopies. Moreover, the activation barrier varies from *E*_*A*_ = 53–62 kJ/mol to 177–192 kJ/mol for measurements carried out below and above *T*_*c*_, respectively (Fig. 5E). This is a quite unexpected result that cannot be explained on the chemical ground. In this context, it should be stressed that generally chemical conversions are described with the use of a single activation energy. It is surely related to the fact that in most cases chemical reactions are carried out in various solvents in low viscosity systems at the relatively narrow range of temperatures.

To understand more precisely the origin of the crossover in the kinetics of mutarotation in L-sorbose, an additional FTIR analysis with the special attention put on the 3000–3600 cm^−1^ region has been performed. The procedure was carried out from two perspectives: the first one is strictly related to the temperature effect on the population of the hydrogen bonds, while the second one is connected to the variation in these specific interactions upon mutarotation carried out above and below the crossover temperature.

**Figure 6:**
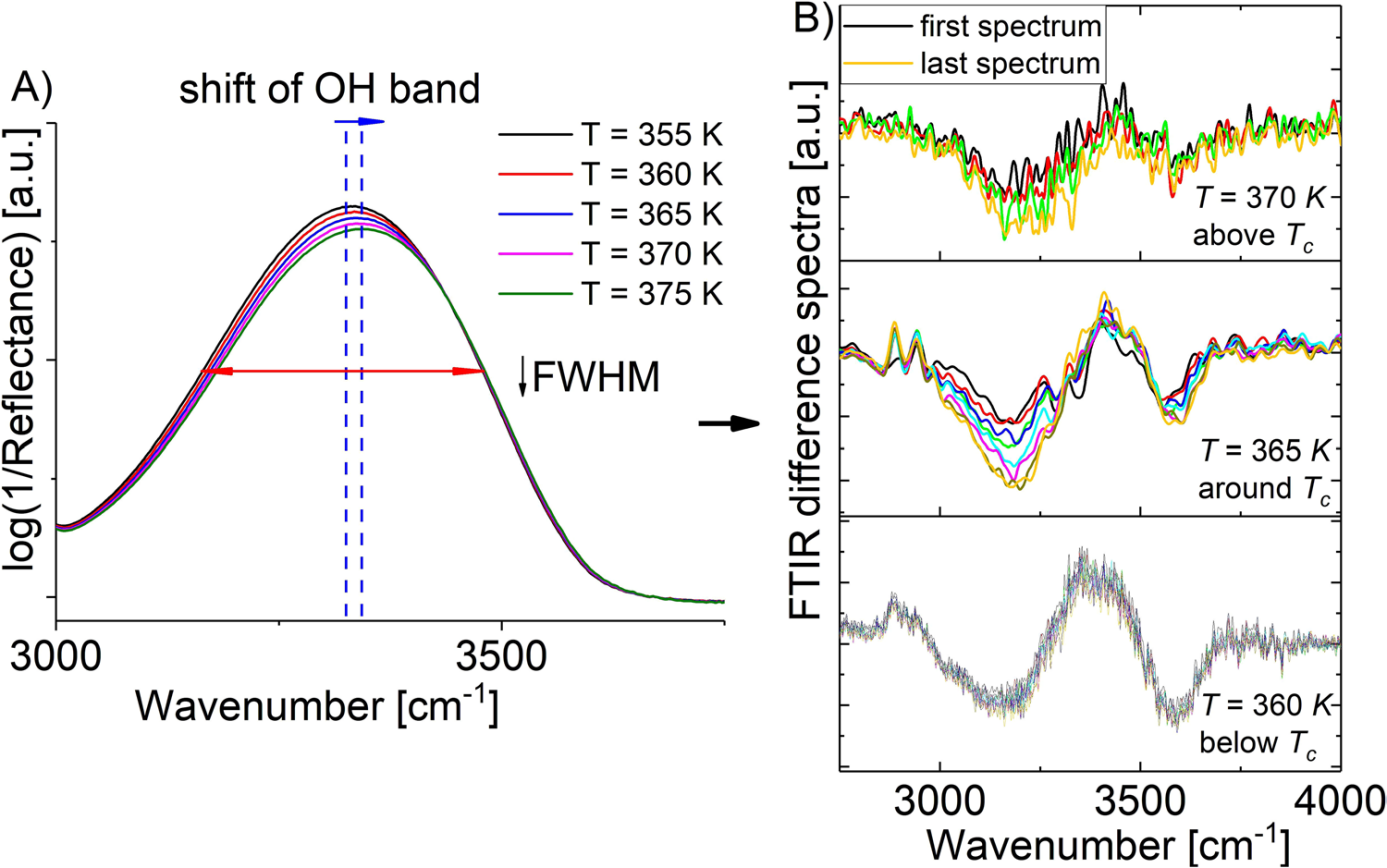
(**A**) General changes within a shift of the OH band position and FWHM (full width at half maximum) during the temperature-dependent experiment. (**B**) The difference spectra analysis of the process performed at selected temperatures: 360 K (below *T*_*c*_), 365 K (*T*_*c*_), 370 K (above *T*_*c*_) over time (for representative spectra only).

As described previously, an increase in temperature is accompanied by the shift of the OH band position towards higher wavenumbers (around 100 cm^−1^) as well as lowering the FWHM (full width at half maximum) (Fig. 6A). These observations suggest a change in dynamics and strength of H-bonds which become more significant above *T* = 365 K. It is surely related to faster rearrangement in the distribution of hydrogen bonds in relation to more viscous and dense system in the vicinity of the glass transition temperature or below *T*_*c*_. On the other hand, the difference spectra analysis obtained for the data collected during mutarotation above and below *T*_*c*_ (*T* = 360, 365, 370 K; Fig. 6B) revealed quite important information about the variation in population and strength of H-bonds. It is worth to mention that this type of data treatment relies on the subtraction of the initial spectra from all of the following in series (difference spectra = FTIR_i_/FTIR_1_, where i = 2, 3, 4,…)^34^. It was found that for the reaction carried out below *T*_*c*_, the maximum of OH band at ~3334 cm^−1^ and two minimums centered about 3600 and 3250 cm^−1^ are practically not affected by the progress of the reaction. It is especially well visible looking more closely at the intensity and FWHM value of such bands which change only slightly over time. This can be an evidence that in the analyzed systems the weak H-bonds are built as a result of (*i*) transformation of a medium hydrogen bonding, (*ii*) dynamics of the system and (*iii*) molecular movements of primarily observed non-hydrogen bonding groups (Fig. 6B). In the case of the reaction carried out above *T*_*c*_ (*T* > 365 K), the time-dependent measurements revealed that the center of *ν*_(OH)_ band is shifted towards higher wavenumbers about ~144 cm^−1^ with respect to the band observed below *T*_*c*_ (Fig. 6B). Additionally, the intensity of this band becomes relatively low resulting in the formation of weaker H-bonds scheme or even formation of non-hydrogen bonding moieties. A weakening of H-bonding pattern visible above *T*_*c*_ might be correlated with the fast movement of molecules due to high thermal energy delivered to the system. Consequently, the proton transfer might be most likely affected leading to significant increase in the activation barrier at *T*_*c*_.

## Conclusion

Inter- and intramolecular dynamics of L-sorbose has been investigated in a wide temperature range. We found that at around *T* = 365 K there is a splitting of structural (*α*) and *γ*-relaxations which are also accompanied by the change in intramolecular dynamics. In this way, the crossover temperature (*T*_*c*_), separating the liquid-like from the solid-like dynamics was determined. Furthermore, studies on mutarotation of L-sorbose with the use of FTIR and Raman spectroscopies were carried out. As support, we performed additional theoretical calculations to assign observed bands to the molecular vibrations in various isomers of carbohydrate and determine the direction of mutarotation. From our data, it is clear that upon reaction α-isomers are formed at the expense of the β ones. It is worth to mention that in this paper we were just focused on the β → α interconversion in L-sorbose. Unexpectedly, it was found that exactly at *T*_*c*_ there is a clear change in the pace of reaction as well as activation barrier for the mutarotation. Such a dramatic change cannot be explained from the chemical point of view. In this context, it is worthwhile to add that usually the progress of this canonical reaction is described with the use of a single activation barrier. Results presented herein indicated unquestionably that the change in dynamics, marking the beginning of the cooperative motions, accompanied by the change in the strength as well dynamics of H-bonds, has a serious influence on the progress of mutarotation. Our investigations can be used to better understand the physics of condensed matter as well as the role of H-bonds in controlling mutarotation in the supercooled state in pure saccharide.

## Methods

L-sorbose (>99% purity) was supplied by Sigma Aldrich and used as received. Dielectric measurements were performed using Novocontrol Alpha analyzer in the frequency range from 10^–2^ to 10^6^ Hz.

Moreover, FTIR and Raman spectroscopies were used to follow molecular rearrangement during mutarotation in a wide temperature range. The FTIR investigations were carried out using an Agilent Cary 640 FTIR spectrometer and GladiATR accessory (Pike Technologies) in the 4000–400 cm^−1^ range and 2 cm^−1^ spectral resolution. Raman measurements were performed using WITec confocal Raman microscope CRM alpha 300 M equipped with a solid-state laser (*λ* = 532 nm). Time-series spectra were accumulated in the 4000–120 cm^−1^ region, with a spectral resolution of 3 cm^−1^.

Quantum mechanical calculations were applied to resolve the problem of band assignments of individual isomers. Density functional theory (DFT) level calculations were done in the gas phase^37–39^ using B3LYP functional^40–42^ and standard split-valence basis sets 6–311++G(2d,2p)^42^. Gaussian09 software package^43^ were used to optimization of the geometry of molecular structures. The optimized structures were taken as input files for vibrational harmonic calculations. All conformers had positive harmonic vibrations proving a true energy minimum^43^. The theoretical methods overestimate vibrational frequencies due to neglecting the anharmonicity, incomplete incorporation of the electron correlation and the use of finite basis sets in the theoretical treatment. Therefore, the spectra were adjusted by a scale factor of 0.976^44^. Theoretical Raman intensities (*I*_*i*_) were obtained from the calculated Raman scattering activities (*S*_*i*_) based on the expression:

5 $${I}_{i}=\frac{{10}^{-12}{({v}_{0}-{v}_{i})}^{4}{S}_{i}}{{v}_{i}{B}_{i}},$$

where *B*_*i*_ is a temperature factor, which accounts for the intensity contribution of excited vibrational states and is represented by the Boltzmann distribution:

6 $${B}_{i}=1-\exp [\frac{(-h{v}_{i}c)}{(kT)}],$$

where *h*, *k*, *c*, and *T* are Planck and Boltzmann constants, a speed of light and temperature in Kelvin, respectively; ${v}_{0}$ is the frequency of the laser excitation line (${v}_{0}=1/{\lambda }_{0}$ where *λ*_0_ is the laser wavelength), *v*_*i*_ is the frequency of normal mode^45,46^.
